# Molecular-trapping in Emulsion’s Monolayer: A New Strategy for Production and Purification of Bioactive Saponins

**DOI:** 10.1038/s41598-017-15067-4

**Published:** 2017-11-06

**Authors:** Titus C. Obasi, Radu Moldovan, Anca Toiu, Cornelia Braicu, Ede Bodoki, Ioana Berindan-Neagoe, Ilioara Oniga, Robert Sandulescu, Radu Oprean

**Affiliations:** 1 Analytical Chemistry Department, Faculty of Pharmacy, Iuliu Haţieganu University of Medicine and Pharmacy, 4 Louis Pasteur Street, Cluj Napoca, 400349 Romania; 2 Center for Functional Genomics, Biomedicine and Translational Medicine, Iuliu Haţieganu University of Medicine and Pharmacy, 23 Gheorghe Marinescu street, Cluj-Napoca, Romania; 3 MEDFUTURE -Research Center for Advanced Medicine, Iuliu Haţieganu University of Medicine and Pharmacy, 6 Louis Pasteur Street, Cluj Napoca, 400349 Romania; 4 Department of Pharmacognosy, Faculty of Pharmacy, Iuliu Haţieganu University of Medicine and Pharmacy, 13 Ion Creangă Street, Cluj Napoca, 400010 Romania

## Abstract

Saponins from defatted root-extract of *Securidaca longipedunculata* were systematically entrapped in emulsion monolayer-barrier and finally recovered in pure form through demulsification. First, their molecules were dispersed in water to engineer a monomolecular film architecture, via self-assembly. Emulsifying with ethyl-ether resulted in swollen micelles and engendered phase-inversion and phase-separation, by disrupting the thermodynamic equilibrium. As positive outcome, a Winsor II system was obtained, having saponin-rich upper phase (ethyl-ether) and impurities bound lower phase (aqueous). Saponin particles underwent transition in insoluble ethyl-ether, precipitated and recovered as solids. The entire process was bioactivity-guided and validated using pooled fractions of securidaca saponins, purified by TLC (RP-C18, F_254_S). TEM and SEM revealed interesting morphologies and particle sizes between nanometer and micron. At the end, purity output of 90% and total recovery of 94% were achieved. Here we show that “molecular-trapping in emulsion’s monolayer” is an effective method for recovery, production and purification of saponins of plant origin.

## Introduction

The protective barrier systems of emulsions and colloids are formed by self-assembly of surfactant molecules around the dispersed droplets, resulting to a monomolecular boundary-film^1–6^. Affirmed by reputable scientific reports, the supra-molecular structures e.g. micelles are product of orderly self-organization of molecules, as proposed by McBain a century ago^7–12^. In liquid dispersion, surfactant molecules assemble spontaneously, forming varieties of structures (aggregates) of different sizes and shapes such as spherical, rod, tubular, lamellar and cubic mesophases. Exploiting these ideas, a new approach was developed for production and purification of bioactive saponins from multi-constituent plant extracts. The mechanism is based on systematic entrapment of molecules in emulsion monolayer-barriers for instant recovery via demulsification. Emulsions and colloids occur as physical change, existing some kinds of weak inter-molecular forces, e.g. hydrogen bond, van der Waals, London dispersion forces etc.^13^. Basically as a physical phenomenon, the self-associated molecules are neither chemically changed nor deformed within the emulsion infrastructural formations. Thus can be recovered intact from emulsion in their most natural forms, without any kind of breakage or addition of new bonds^14–19^, while retaining inherent biological properties. Debuting first in the field of separation science, this approach is being proposed as pragmatic intervention to age long challenges in the saponin bioprocessing industry, which has constituted serious nightmare. Now with the new innovation, the process of recovery of such target product is simplified into two major steps; first by emulsification and followed by demulsification. Usually, the former is a household term in food and pharmaceutical processing, while the latter constitute a general practice in petroleum and gas industry for separation of oilfield emulsions^20^. For many decades, such has been used in sustaining crude oil production, yielding as bye-products some naturally occurring surfactants such as asphaltenes^21^, resins, oil-soluble organic acids, naphthenic and carboxylic acids, etc.

This new approach however was realized using a dried defatted root-extract of *Securidaca longipedunculata* as emulsifier’s feedstock for solubilizing a non-polar organic solvent, ethyl ether in water. Here the saponin molecules in the feedstock have the functional role as emulsifier, while coexisting together in the mixture of many unwanted compounds. Belonging to class of natural emulsifiers, saponins are primarily classified as large molecular weight glycosides of plant secondary metabolites. Their presence have been reported in more than 100 families of plants, as well as few marine resources^22^. Evidences have shown that saponins are very rich in *S. longipedunculata*, first reported by Lenz in 1913 when he found about 0.94% of neutral saponin in the root bark^23^. In 2010, Mitaine-Offer and co wokers^24^, isolated four new acylated triterpene bidesmosides, making a total of eight saponins compounds already found in the plant. Saponins are characterized by detergent, foaming and hemolytic properties^25,26^, while possessing in a single molecule a polar hydrophilic and non-polar hydrophobic groups. Usually a hydrophobic core of C27 steroidal or C30 triterpenoid aglycone (sapogenin) is esterified or conjugated to monosaccharide units^27^. Their biological and physicochemical properties are often a reflection of vast and complex structural diversity. In liquid dispersions, they form micelles at very low critical micelles concentration (CMC)^25,28,29^, a character which mostly differ from one surfactant molecule to another, depending on the molecular weight, structural arrangement and hydrophile-lipophile balance (HLB)^30,31^, etc.

In nutshell, the mechanistic principle of this new approach encompasses the following: i) dispersion and entrapment of molecules in monolayer boundary-film via self-assembly; ii) emulsification and solubilizing of organic solvent to form an enlarged droplet with amplified liquid core surrounded by surfactant monolayer iii) demulsification, which outcome brings about phase-separation and precipitation of solutes in insoluble organic phase, passing through glassy state transition or kinetically frozen micelles^32^. Similar situation have been reported using co-solvents and solvent evaporation methods^33^. Antisolvents such as ethyl ether and acetone have equally been used to precipitate saponins and other glycosides from miscible solutions in nonselective manner^22,34^. Here, emphasis is laid on importance of self-assembly as tool for separating, purifying and isolating bioactive constituents from plants, with minimal consumption of solvent and less damage to the desired molecules. For the past few decades, several scientific innovations have been built on the principles of molecular self-assembly. In the area of healthcare for instance, some spherical-shaped self-assembled nanoparticle have been designed for delivering active drugs and devices into the remote body organs. The same way, some studies have equally shown that self-assembled peptides and protein-based nanomaterial are becoming quite significant in cancer and oncology, as recently demonstrated in photothermal and photodynamic antitumor directed therapy^35–37^.

## Results

### Recovery of Saponins from demulsified emulsion

**Figure 1:**
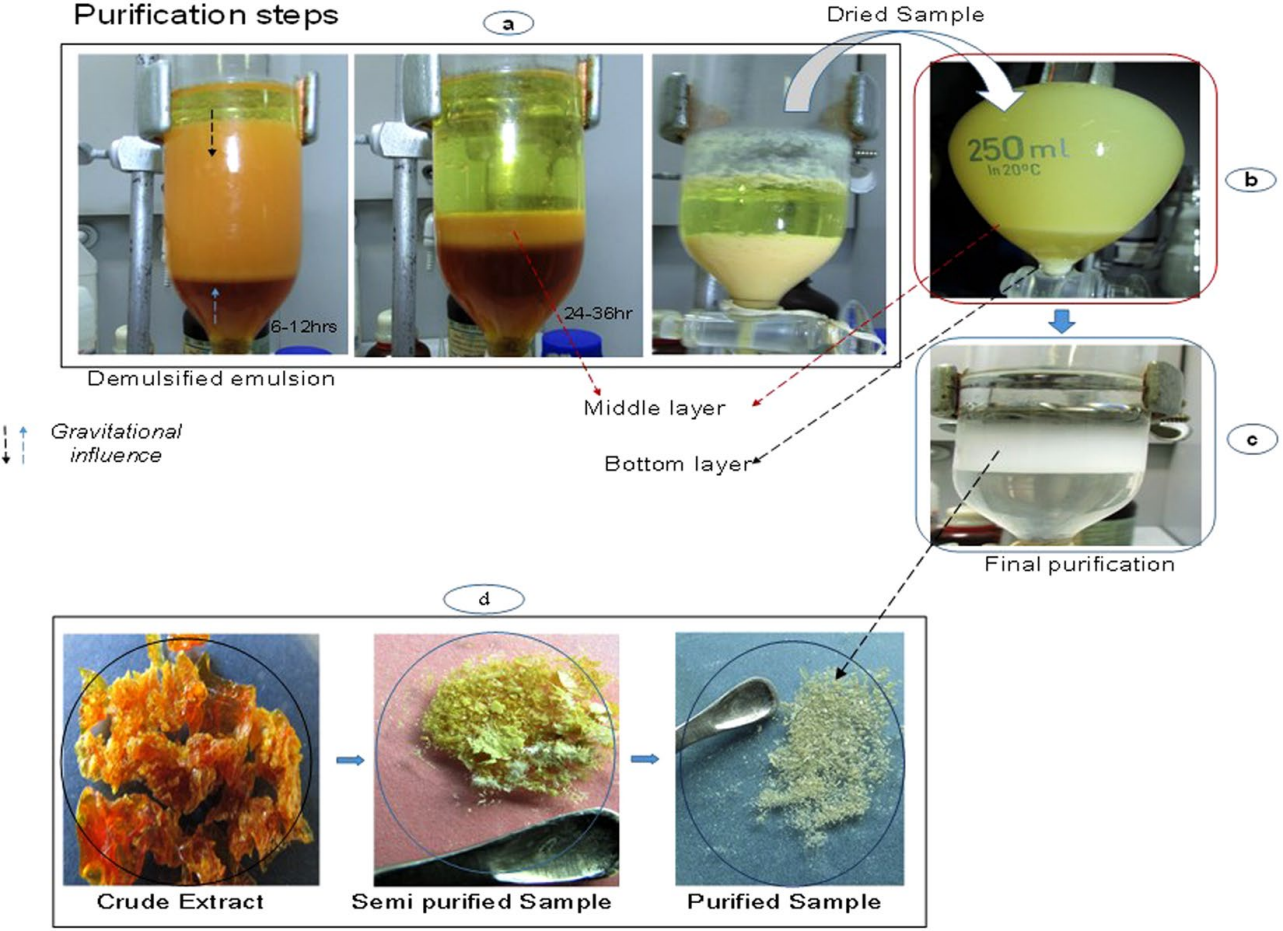
Production and purification steps: (**a**). The primary recovery of 4 A; (**b**) Intermediate purification step (cycle 1) showing gradual sedimentation at both middle and bottom; (**c**) Final purification step (cycle 3); (**d**) the dried extract, semi-purified and purified saponin (4A).

**Figure 2:**
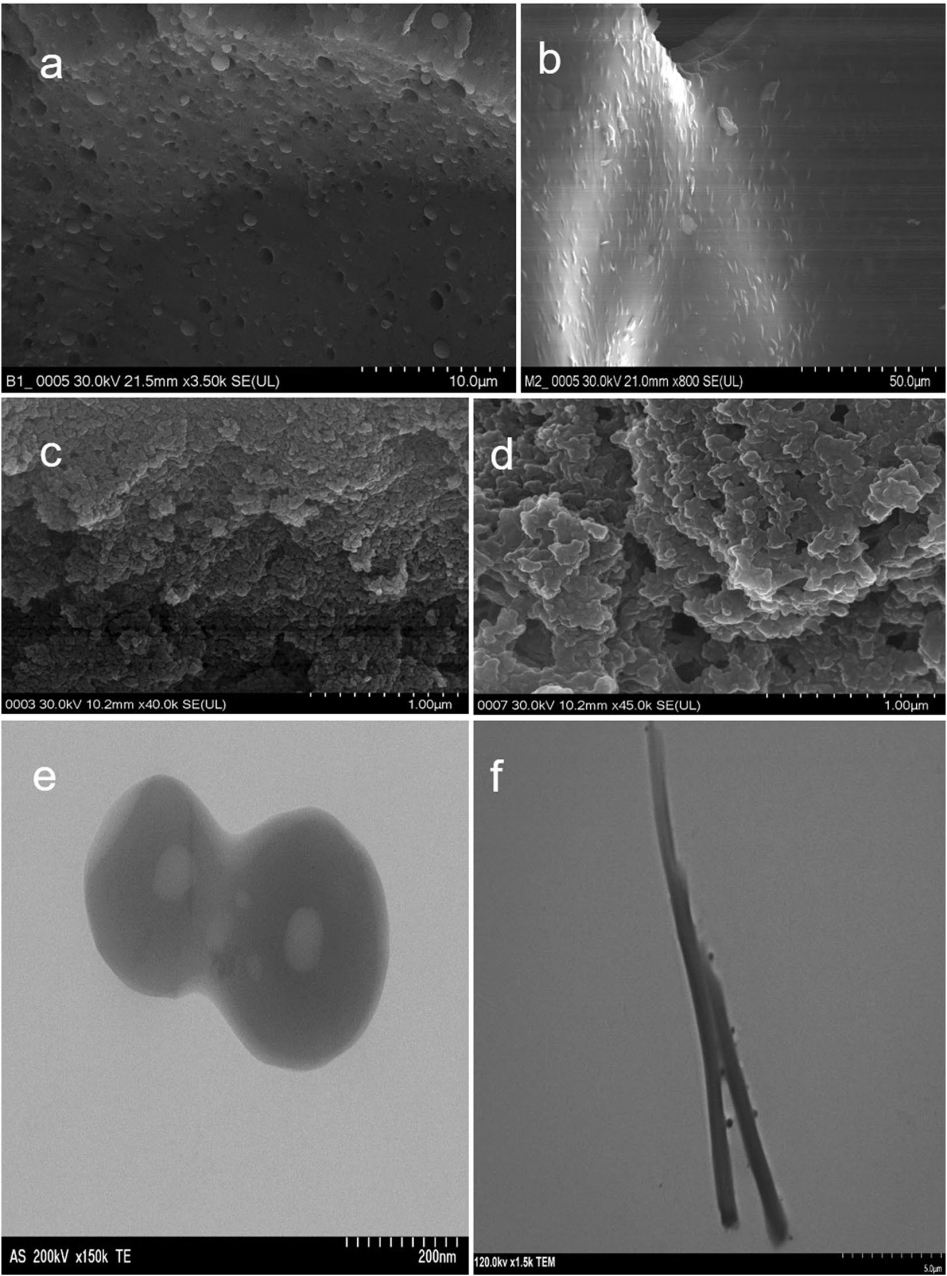
Morphological characters by TEM and SEM micrograph. Images showing the microscopic character of particles found in the demulsified emulsion: (**a**) The bottom layer; (**b**–**d**) the middle layer; while (**e**,**f**) the artifacts (emulsion infrastructures). Particles in the bottom layer are in pellet form, which could be useful in their nano dimension as vehicle for drug transport, proteins and immunotoxins target delivery.

**Figure 3:**
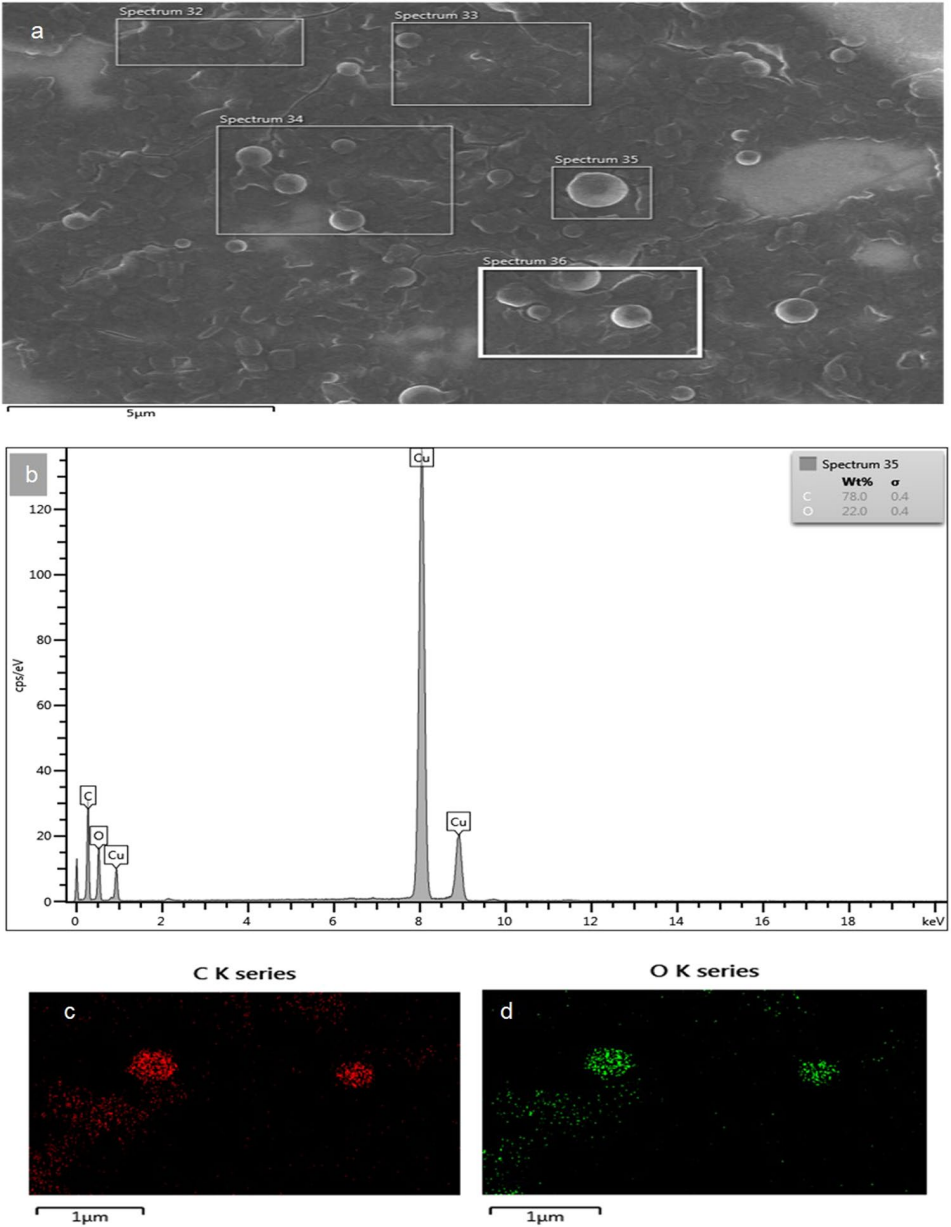
Elemental analysis by energy-dispersive X-ray spectroscopy (SEM/EDS): (**a**) EDS X-ray mapping showing electron images of spherical 4A particles. (**b**) EDS spectrum showing the percentage elemental compositions in 4A particle (Spectrum 35). The primary peaks suggest a typical hydrocarbon by the ratio of carbon and oxygen. Usually, hydrogen cannot be observed by X-ray spectroscopy; (**c**,**d**) showing respective EDS/electron distribution of carbon and oxygen on the surface of 4A particle. Here the colors of red (**C** K series) and green (**O** K series) represent carbon and oxygen respectively.

The dried methanolic root-extract of *Securidaca longipedunculata*, acted as surfactant/emulsifier by enhancing the emulsification (i.e. solubilization) of ethyl-ether in water. The recovery of saponins was possible in relatively pure state, after the emulsion has been destabilized. Saponin materials were deposited at the middle and bottom layers of destabilized emulsion [Fig. 1(a–c)], in varieties of shapes. TEM and SEM revealed different morphologies and particle sizes, ranging from few microns to nanometer scale as shown in “Fig. 2”. Elemental analysis by energy-dispersive X-ray microanalysis (SEM/EDS) further revealed the percentage composition of carbon and oxygen in the particles [Fig. 3]. No heavy metal was detected to suggest any kind of complexation reaction or chemical precipitation. Demulsification was supported by phase-separation, which is secondary to phase inversion from oil-in-water (o/w) to water-in-oil (w/o) emulsion. The type of separation observed, depicted Winsor II phase-equilibrium, whereby the emulsifiers substances are saturated in the upper organic phase, in what seemed like creamy suspension and very turbid. There was observable sedimentation of particles in the upper phase, which further enhanced the recovery of saponins. The purity of the finished product labelled 4 A was tremendously improved in three successive purification cycles, likewise the physical appearance. Ethyl ether behaved as suitable antisolvent medium which promoted the precipitation and sedimentation of particles in the upper organic phase as shown in “Fig. 1(a–c)”.

### TLC/Chemical characterization

4 A produced nine different fractions on reversed-phase TLC separation. Fractions 1–5 reacted positively to saponin identification tests as found in “Supp. Table S1”, while the rest failed and hence classified as impurities. The TLC fractions (2, 4 and 5) show characteristic blue color under UV 365 nm, while (1 and 3) have none. Blue-violet color was seen only after derivatization reaction with 10% ethanolic sulphuric acid and heat at 105 ^o^C [Supp. Table S1]. Heating for about 3 minutes, fractions (1 and 3) showed blue-violet color under UV 365 nm, while further exposure up to 6 minutes produced intense brown coloration instead. The most dominant fraction (i.e. TLC fraction no. 4) occupied over 50% v/v of the entire chromatogram.

### Quantification of impurities by UV-Vis spectrophotometer

**Figure 4:**
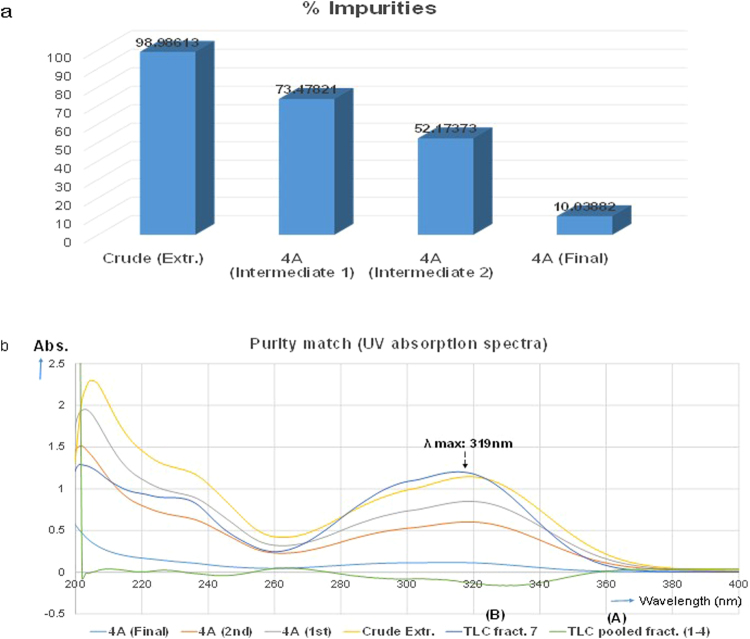
The level of impurities (measured by UV-Vis spectrophotometry): (**a**) Percentage impurities: showing that 4A (final) has the least impurities compared to intermediate products 1^st^ and 2^nd^ cycles. Regression curve (y = 9.2813x − 0.0028) and R² = 0.9999; (**b**) Purity match: comparing the purities by matching the spectral lines.

The 4 A samples exhibited poor absorption of UV signals within the range of 200–400 nm in much closer relationship with improvised standard A. In contrast, the standard B which is a purified (TLC fraction 7) suspected as phenolic compound, exhibited a closer relationship with the crude extract but not with standard A. While A is a pooled fractions of saponins purified by TLC, they exhibited negative signals due to poor absorbance and inherent lack of chromophore [Fig. 4b]. The overall outcome show that the level of impurities in the crude extract was drastically decreased as a result of purification, shown in “Fig. 4a”.

### Validation of concept

To further confirm our conclusion, an emulsion was formed with water and ethyl ether using a pooled fractions of TLC-purified securidaca saponins. The outcome appeared translucent, replicating exactly the same pattern as previously experimented. In addition, a saponin deposit was formed in the creamy middle layer. The amount of recovery achieved in the first purification cycle was slightly above 50% (w/w) [Supp. Fig. S1]. The total yield after the fifth continuous cycle were summed up to 94% approximately. The alternative experiment with classical anionic surfactant (sodium lauryl sulphate) produced similar behavior, but no practical yield of solid deposits at the middle.

### The Critical Micelle Concentration (CMC)

The value of CMC determined at room temperature (25 ^o^C) was 0.033 ± 0.001 g/L, shown in the chart “Supplementary Figure S3b”. This value was derived from the plot of absorbance against the log concentration of 4 A. At the onset of solubilization, a sudden rise in absorbance occurred as micelle aggregates began to form. Two peak wavelengths were initially detected in n-hexane dye solution at 320 nm (UV) and 500 nm (visible). The former was essential, being retained in the solubilized aqueous 4 A medium, unlike the visible range that disappeared on the course of solubilization.

## Discussion

Plant extracts are usually a mixture of closely and non-closely related compounds with varying disparities in physical, chemical and physicochemical properties. Such differences are often very important and should be identified as targets for essential separation and recovery of individual constituents. Here the disparity in CMC values or Krafft temperature is considered paramount, as we face a multicomponent mixture with relative individual surfactant properties^25,28^. For natural nonionic surfactants like saponins, the CMC values are always very low^28^, being an indicative sign of comparative advantage over several other competing chemical species in coexistence. In 4 A for instance, the value of CMC was found at such a very low concentration as 0.033 ± 0.001 g/L [Supp. Fig. S3b], perhaps due to the nature of constituent molecules and, or the function of purity^38^. As a matter of fact, CMC is seen as one of the primary factor driving the segregation and selective accumulation of desired molecules within the monolayer apparatus^39^. It controls and modulate the mechanism for effective separation of chemical species right at the molecular level [Supp. Fig. S3]. In terms of behavior, the molecular entities forming the aggregates are always in equilibrium and can neither be held kinetically static nor physically isolated from the bulk medium, unless by special means e.g. emulsification, demulsification, etc. The reason being that the associated molecules in quasi fluid mosaic state are usually in constant interaction with the free monomers within the bulk medium^39,40^. Generally for emulsions, the thermodynamic equilibrium can be shifted, when the size of the vesicles (droplets and swollen micelles) are exceedingly enlarged, resulting to emulsion instability, phase separation/transition^41–43^ etc. Such activities can bring about the precipitation of polymeric emulsifier materials from emulsion, with chances of enhanced recovery. This is confirmed by the pattern of separation observed in [Fig. 1] and [Supp. Figure S1b], which demonstrate high level of consistency with the prescribed mechanism as shown in “Supplementary Figure S3”. This is also true for the results shown in [Fig. 4], in terms of effectiveness and purity. Saponins are deficient in chromophore and accordingly, the purified 4 A maintained a poor absorption spectra in much closer agreement with standard **A (**pure saponins) obtained by TLC, but not with standard **B** nor the crude extract [Fig. 4b].

In aqueous dispersion, saponins are far more soluble in water than in most organic solvents, thus favoring oil-in-water emulsion, in line with Bancroft rule^44^. To get rid of impurities in the extract, it was necessary to inverse the phase of the emulsion from o/w to w/o system, relying on the fact that both emulsifier (4 A) and the impurities are soluble in water (the continuous phase). In total agreement with Winsor type II phase-equilibrium^45^, the 4 A particles (saponins) were selectively saturated in the upper organic phase, while deficient in the lower phase, extensively filled with water and soluble impurities [Fig. 1a]. By saturating the saponins in relatively insoluble solvent like ethyl-ether, spontaneous precipitation could be triggered. The different shapes and sizes of particles observed by TEM and SEM [Fig. 2], suggest a possible clue for the particles origin, which perhaps could be linked to emulsion infrastructure. In addition, the outcome of demulsification into four compartmentalized divisions - (the upper oil phase, creamy emulsifier-rich middle, aqueous phase and pellets at the bottom) as shown in [Supp. Fig. S1b], further reveal a positive conformation with other notable systems of destabilized emulsions^14–17,46–49^. Within this position, it might be justified to predict that the origin of the small fragmented flat sheets seen in [Fig. 2d] may not be far from the ruptured monolayer films. Relevant analogy could be drawn from the transition and evolution of o/w to w/o system, which affords the chance of frequent passages through lamellar phases e.g. (o/w -~ L~--, w/o)^39^, forming varieties of shapes and extended flat lamellar (Lα or L3 isotropic structures). The smectic liquid crystalline observed in [Fig. 2b] may also serve as relevant example. Further experimental results may be useful to form more concrete evidence, as this might be necessary to establish the evolutionary trend or show possible transition from normal micelles to swollen micelles, and finally to particles in glassy state (kinetically frozen micelles). During glassy phase transition, the self-organized molecules are firmly trapped in kinetically locked state, within their fixed formation in the monolayer architecture, leading to physical transformation. Quite related but not entirely same, Nagaranjan and others^40,50,51^ have reported the favorable criteria for glassy state transition, which might also be applicable here, according to Chow’s thermodynamic model. Finally, the main objective of this research however has been fulfilled, being able to recover from plant a purified saponin in much selective manner and greater efficiency in terms of value addition than ever attained in the history of saponin production and purification, using ordinary solvent-solvent method. Having evidence of high antiproliferative effects, low IC_50_ and outstanding selectivity index against tumor cells [Supp. Fig. S2(b,c)], some 4 A products can easily find application in cancer therapy. They can equally be used as adjunct for vaccines, ISCOMs or serve as vehicle for drug targeted delivery based on their ability to form spherical particles and shapes on self-assembly [Fig. 2a]. We are quite convinced that simple chemical characterization tests used in confirming the suspected phytochemical groups as in Supp. Table S1 are very essential. Much importantly because, they target specific colors resulting from the increasing chemical conjugation and unsaturation in the sapogenin aglycone ring(s). Though might exist some minor limitations, the method is quite useful in providing relevant data for further investigations. Based on the above, further studies have been scheduled to elucidate the molecular structure of the individual components of 4 A using NMR and Mass spectrometry, etc. The main purpose is to identify, ascertain and correlate the relationship between the nature of the individual molecules, the position of sugar linkages and the type of particles or shapes predominantly formed on self-assembly.

## Methods

### Production and purification of saponins from plant extracts

100.050 g of pulverized root-powder of *Securidaca longipedunculata* was thoroughly defatted with 500 ml of dichloromethane (DCM) for 24 h, using Soxhlet apparatus to remove the undesirable lipids. Residual solvent in the marc was totally removed by air drying before re-extracting with pure methanol for 48 h. A residue of 12.612 g was recovered under reduced temperature in a rotary evaporator. About 5.045 g of the residue was completely dissolved in 200 ml of distilled water in a flask and dispersed for 30 minutes in ultrasonic bath. The resultant aqueous mixture was then mixed with 400 ml ethyl-ether with vigorous agitation to form oil-water emulsion. The content was transferred into a separating funnel and allowed to stand at room temperature. Within 3–6 h phase-separation occurred, forming a creamy precipitate in the upper phase (ethyl-ether), while leaving a reddish-brown clear solution below (aqueous phase). Sedimentation continued in the upper phase, until a thick solid layer is resulted at the middle interface between organic and aqueous phase [Fig. 1]. At the end of 24 h, the middle layer and the few particles also found at the base were carefully collected. Supernatants liquids removed by centrifugation at low speed 14.5 rpm. A total yield of 0.7 g cream colored material was obtained. This was purified further in three cycles, repeating same procedure to realize the final product labeled 4 A. Confirmatory test for saponin was carried out (Liebermann-Burchard and foam tests). The particles were observed under high resolution transmission and scanning electron microscopy (TEM and SEM) to evaluate their morphologic and microscopic characters. Elemental analysis was determined by SEM/ Energy-dispersive X-ray spectroscopy (SEM/EDS). The entire process was bioactivity-guided, as each step underwent appropriate antiproliferative screening (MTT assay) against cervical cancer cell line Caski [Supp. Figure S2b].

### TLC/Chemical characterization of 4 A

Reverse-phase TLC was used to separate the composition of 4 A. At analytical stage, the mobile phase composition MeOH/water/formic acid (4:1:0.1) was selected and optimized, using pre-coated glass backed RP-C18, F_254_S silica gel plates (Merck, Darmstadt, Germany). Saponin spots were visualized under UV 365 nm by modified method of Fenwick and Oakenfull^52^, after spraying the plate with 10% (v/v) H_2_SO_4_ ethanol solution (analytical grade). With the optimized conditions, preparative TLC was conducted using a 2 mm, 20 × 20 mm glass plate. The plate was developed in a conventional 20 × 20 mm glass twin through chromatographic chamber (CAMAG, Muttenz, Switzerland) at room temperature (~22 ^o^C) and ~60% relative air humidity. Fractions were scrapped and eluted with methanol to recover the solutes. Isolated fractions of 4 A (1–5) that responded positively to preliminary saponin test, were further subjected to derivatization reactions with Carr Price and Liebermann-Burchard tests, as well as thymol-H_2_SO_4_ reaction on the acid (HCl) hydrolyzed portions. Saponin spots were visualized under UV at 365 nm after heating between 105 and 110 ^o^C for 3–6 minutes.

### Quantification of impurities by UV-Vis spectrophotometer

Two improvised standards A and B were prepared from 4 A by purifying with reverse-phase preparative TLC. A is a pooled fractions of securidaca saponins, comprising the pure TLC fractions 1–4 that tested positive to saponin confirmatory tests as in [Supp. Table S1], while B is the TLC fraction 7, which tested negative. Double beam UV-Vis spectrophotometer (Specord 250 Plus, Germany) was used to determine the absorbance spectra of the analyte dissolved in methanol on the range of 200 nm and 400 nm. Whole spectra was run and calibrations determined at the peak wavelength (319 nm) of the crude extract on serial dilutions from 0.25 mg/ml to 0.0078 mg/ml. Since methanol is the solubilizing liquid, a pure methanol was used as reference. The analytes include: i) the product 4 A (final); ii) the crude extract; iii & iv) the partially purified intermediates 4 A (1^st^ and 2^nd^) cycles respectively. Before taking the measurement, a reference standard was initially run with solvent (MeOH) only. All absorbance measurement were performed in triplicate (n = 3).

### Preliminary validation of concept

Two improvised standards of purified securidaca saponins, designated as fractions (2 and 4) derived from reverse phase preparative TLC of 4 A were pooled together and used as emulsifier. The fractions were previously characterized, confirming the presence of triterpenoid saponins [Supp. Table S1]. Emulsion was prepared by strictly adhering to the procedures and conditions as described  earlier. Here, 50 mg of the pooled fractions was used, while the volume of water was adjusted to 100 ml. Afterwards the completion of phase-separation in 24 h, the saponin-rich materials were collected and accurately weighed. The resultant aqueous solutions were successively emulsified further until the fifth cycle, to recover all the remnant saponins in the solution. The experiment was reproduced in triplicates (n = 3) recording the average weights of recovered samples. Similar process was carried out using an anionic surfactant, 50 mg of sodium lauryl sulphate (SLS), sourced from Merck, Germany. SLS was used as emulsifier, applying the same conditions as above.

### Determination of Critical Micelle Concentration (CMC)

Dye solubilization technique was employed, using an aqueous insoluble dye, 1-[(E)-{4-[(E)-Phenyldiazenyl] phenyl} diazenyl]−2-naphthol (Sudan III) in a modified method, applied in S. G Verza *et al*. 2012. A solution of Sudan III in n-hexane (1 mM) was first prepared and the peak wavelengths established using a double beam UV-Vis spectrophotometer (Specord 250 plus). The CMC was assayed by adding 200 µL of dye solution to 5 ml volume of a serially diluted aqueous 4As at the following concentrations - (0.25, 0.1, 0.05, 0.01, 0.005, 0.0005 0.0001) g/L. The resultant mixtures were vigorously shaken and allowed to stand for 30 min. Aliquot from the lower phase of each mixture (3 ml) was carefully transferred into a cuvet and the absorbance readings determined at (320 nm). The experiment was performed at room temperature of 25 °C. Before taking measurements, a blank solution of the diluents (distilled water) was first of all run as reference. All measurements were made in triplicates and absorbance plotted against the logarithm of concentration. At concentrations below CMC, dye appears insoluble and the absorbance reading maintained near zero, but once the micellar aggregates began to form, the dye solubilizes, resulting to a sharp rise in the value of absorbance.

### Bioactivity Screening

#### ***C***ell culture

Two cell lines were used, i) cervical cancer cells (Caski) and ii) fibroblast cells (HFL). Caski cells were cultured in Gibco RPMI 1640 Medium 1X, supplemented with 10% fetal bovine serum (FBS - Gibco) and 1% Penicillin-Streptomycin. For HFL1, we used 15% fetal bovine serum (FBS - Gibco), 1% Penicillin-Streptomycin (Sigma), 2 mM glutamine (Gibco), MEM-NEAA (MEM Non-Essential Amino Acids Solution (100X) - Gibco) and DMEM Medium 4.5 g/L D-glucose (Sigma)/Nutrient Mixtures F-12 HAM (Sigma) (v 1:1). Cell lines were maintained at 37 °C and 5% CO2 in a humidified incubator.

#### Cell proliferation evaluation by MTT test

Cell proliferation and IC50 were measured by MTT assay using multiple doses of the crude extracts, 4 A and the nine TLC fractions, on the aforementioned cell lines. The MTT salt is cleaved by dehydrogenase in the living cells and reduced to an insoluble formazan crystal which shows a specific purple color. The color was detected by a plate reader (Synergy H1 Hybrid Reader Biotek). Caski and HFL1 cell lines were seeded overnight in 96-well plate at an 8 × 103 cells/well density. After incubation, cells were treated with corresponding compounds dose and incubated for another 24 hours. Cell suspensions were removed, added 150 μl of MTT solution and incubated further for 1 hour. The solution was removed and added 100 μl DMSO added to dissolve the formazan product and incubated for about 5–10 minutes in a plate shaker. After the incubation, the absorbance was measured at 570 nm in a plate reader (Synergy H1 Hybrid Reader Biotek).

### Data availability

The raw data used to generate the figures and tables in this manuscript are available from the corresponding or the first author upon request.

## Electronic supplementary material

Supplementary Information
