# Supplementary material for: Molecular-trapping in Emulsion’s Monolayer: A New Strategy for Production and Purification of Bioactive Saponins
Source: Sci Rep. 2017 Nov 6;7:14511. doi: 10.1038/s41598-017-15067-4 (PMC5674058; doi:10.1038/s41598-017-15067-4)
Supplement: Supplementary file 1 — Supplementary Information [file 41598_2017_15067_MOESM1_ESM.pdf]

Supplementary Information

Molecular-trapping in Emulsion's Monolayer: A New Strategy for Production and Purification of Bioactive Saponins

Titus C. Obasi[1], Radu Moldovan[1], Anca Toiu[4], Cornelia Braicu[2], Ede Bodoki[1], Ioana Berindan-Neagoe[2, 3], Ilioara Oniga[4], Robert Sandulescu[1], Radu Oprean[1*]

[1]Analytical Chemistry Department, Faculty of Pharmacy, Iuliu Haţieganu University of Medicine and Pharmacy, 4 Louis Pasteur Street, Cluj-Napoca (400349), Romania

[2]Center for Functional Genomics, Biomedicine and Translational Medicine, Iuliu Haţieganu University of Medicine and Pharmacy, 23 Gheorghe Marinescu Street, Cluj-Napoca (400337), Romania

[3]MEDFUTURE -Research Center for Advanced Medicine, Iuliu Haţieganu University of Medicine and Pharmacy, 6 Louis Pasteur Street, Cluj-Napoca (400349), Romania

[4]Department of Pharmacognosy, Faculty of Pharmacy, Iuliu Haţieganu University of Medicine and Pharmacy, 13 Ion Creangă Street, Cluj-Napoca (400010), Romania

*Correspondence should be addressed to Radu Oprean; roprean@umfcluj.ro

Activity/items	4A	4A1	4A2	4A3	4A4	4A5
Foam test	+++	+++	+++	+++	+++	+++
Color	Cream ^{v}	Nil ^{v}<br>Nil ^{uv}	Nil ^{v}<br>Blue ^{uv}	Nil ^{v}<br>Nil ^{uv}	Nil ^{v}<br>Blue ^{uv}	Nil ^{v}<br>Blue ^{uv}
10% EtOH-H_2SO_4 (@105 deg C; 3min.)	Yellow ^{v}	Nil ^{v}<br>Violet ^{uv}	Yellow ^{v}<br>Yellow ^{uv}	Nill ^{v}<br>Violet ^{uv}	Yellow ^{v}<br>Yellow ^{uv}	Yellow ^{v}<br>Yellow ^{uv}
10% EtOH-H_2SO_4 (@105 deg C; 6min.)	Yellow ^{v}	Greenish-yellow ^{v}<br>Brown ^{uv}	Greenish Yellow ^{v}<br>Yellow ^{uv}	Dark-green ^{v}<br>Brown ^{uv}	Dark-green ^{v}<br>Yellow ^{uv}	Dark-green ^{v}<br>Yellow ^{uv}
Lieberman test.	Pinkish (ring) ^{v}	Yellow ^{v}<br>Pink ^{uv}	Yellow ^{v}<br>Yellow ^{uv}	Slight-pink ^{v}<br>Pinkish ^{uv}	Pinkish ^{v}<br>Yellow ^{uv}	Faint ^{v}<br>Bluish ^{uv}
Carr Price test		Slight-pink ^{v}<br>Pink ^{uv}	Slight-pink ^{v}<br>Slight-yellow ^{uv}	Slight-pink ^{v}<br>Pink ^{uv}	Purple^{v}<br>Blue-violet^{uv}	Faint^{v}<br>Blue-Violet ^{uv}
Thymol-H_2SO_4		Purple ^{v}<br>Violet ^{uv}	Purple ^{v}<br>Violet ^{uv}	Purple ^{v}<br>Violet ^{uv}	Purple ^{v}<br>Violet ^{uv}	Purple ^{v}<br>Violet ^{uv}
Suspected class		Triterpene saponin	Triterpen saponin	Triterpene saponin	Triterpene saponin	Triterpen saponin

Key: (V) Visible light; (UV) Ultraviolet light 365nm

Supplementary Table S1. Chemical characterization and derivatization reactions. 4A is a purified saponin, while 4A (1-5) are different TLC fractions.

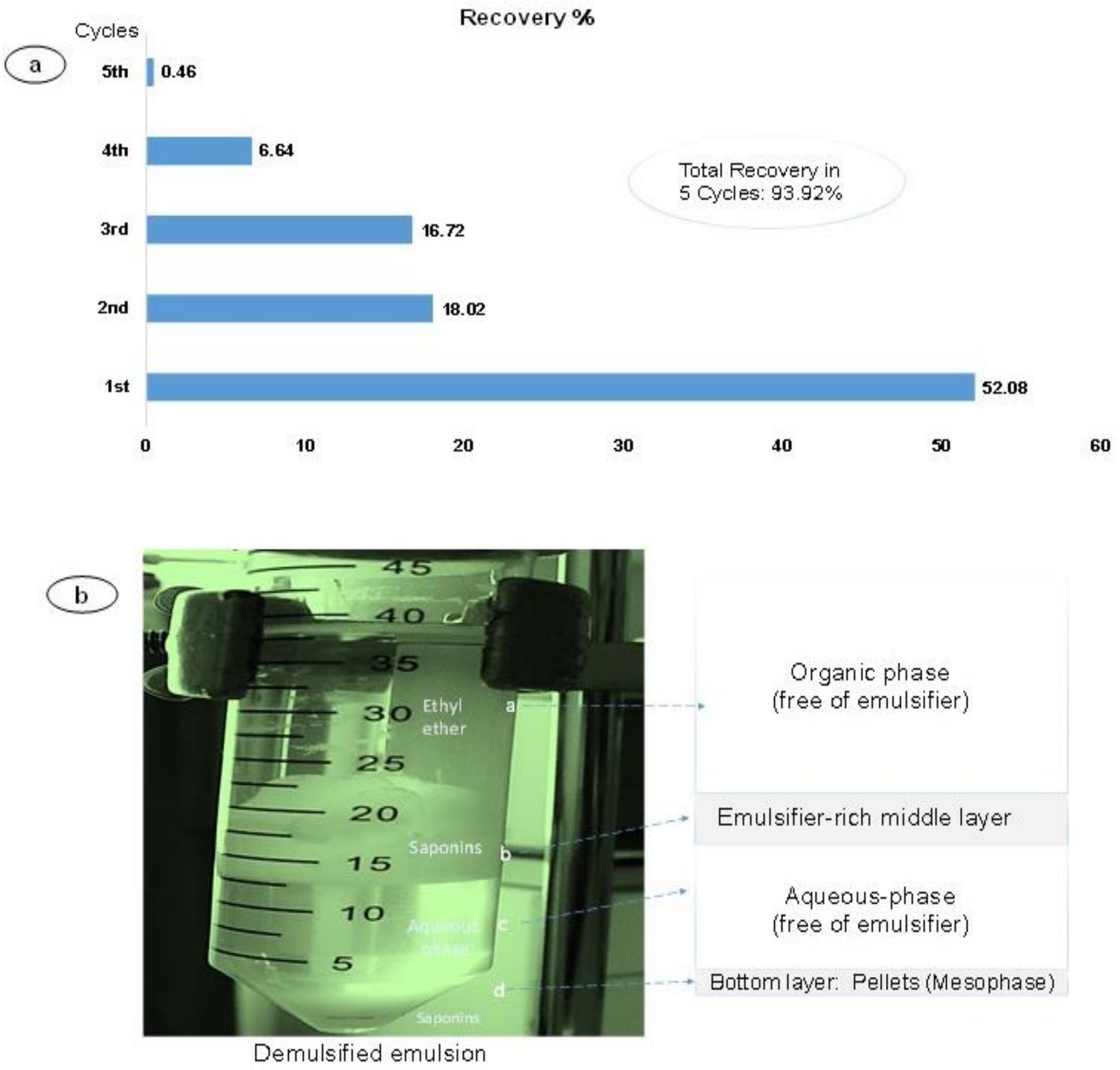

Supplementary Figure S1. Validation experiment. **a**) The chart showing percentage recovery from pooled fractions of pure securidaca saponins, with total yield of 93.92%. **b**) The classical products of complete destabilized emulsion, appearing in four phases: an oil phase, creamy emulsifier-rich middle phase, an aqueous phase and pellets (liposomes and mesosphere).

a

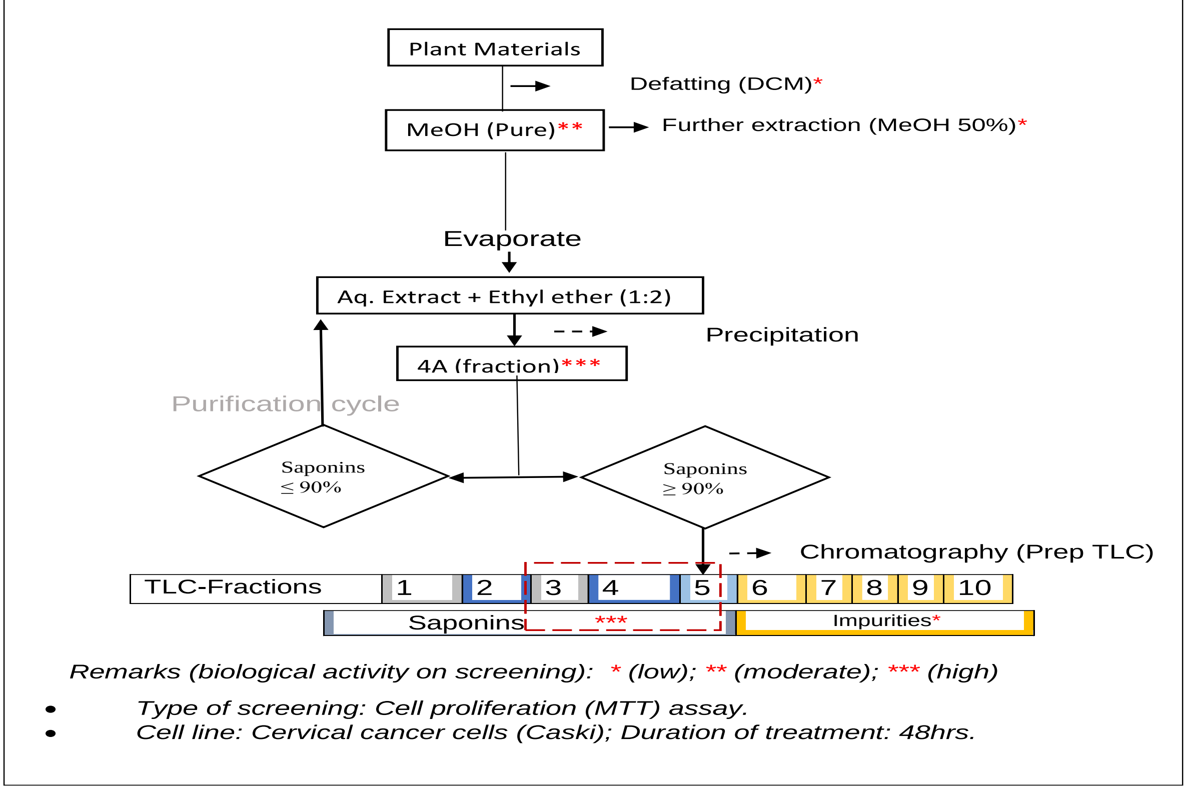

b

Extracts / Fractions	Antiproliferative activity
Control 1 (H_2O)	93.76±0.93
DCM	60.41±3.77
MeOH	7.64±0.26
4A	5.23±4.53
MeOH-H_2O (50%)	96.81±2.51
Control 2 (EtOH 10%)	96.94±3.28

c

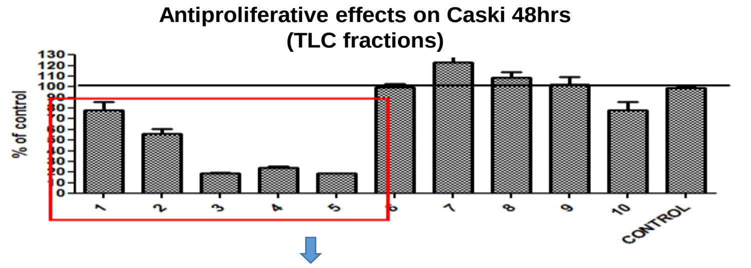

Time (hours)	TLC fractions	Caski cells	HFL cells	Selectivity Index (SI)
		IC_{50} µg/ml	IC_{50} µg/ml	
24	3	0.02282	0.006078	0.0266
	4	0.04540	0.351200	7.7357
	5	1.297	46.64	35.959
48	3	0.02502	0.013460	0.5379
	4	0.04833	2.845	58.866
	5	~ 71.88	~ 71.88	-

Supplementary Figure S2. Bioactivity-guided separation and purification. a) Process scheme; b and C) Biological activities on screening.

a

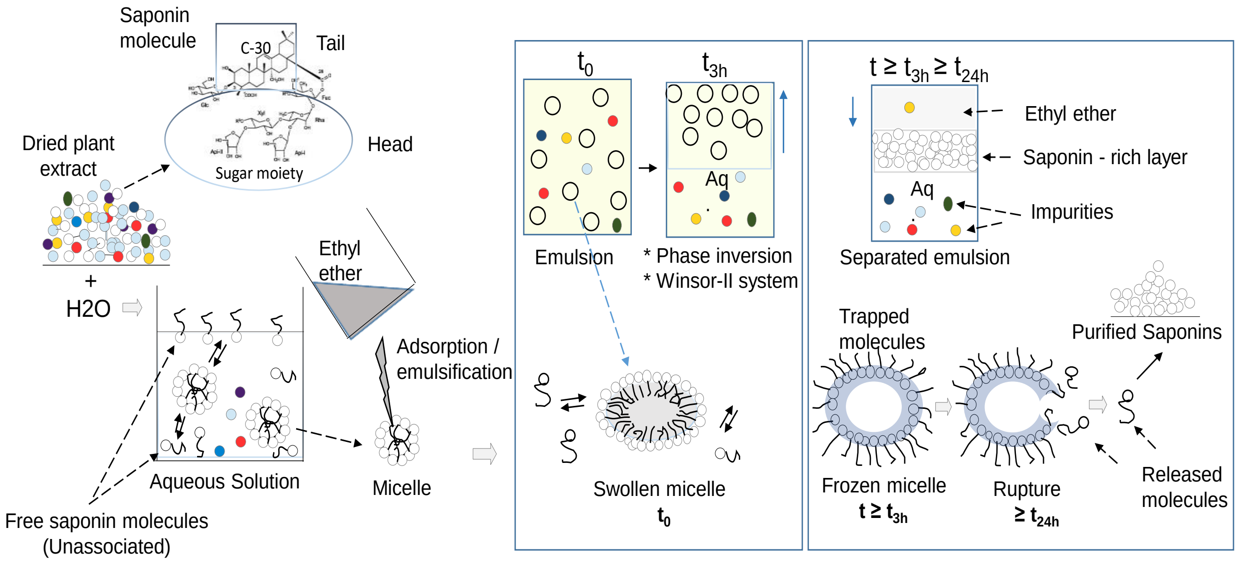

b

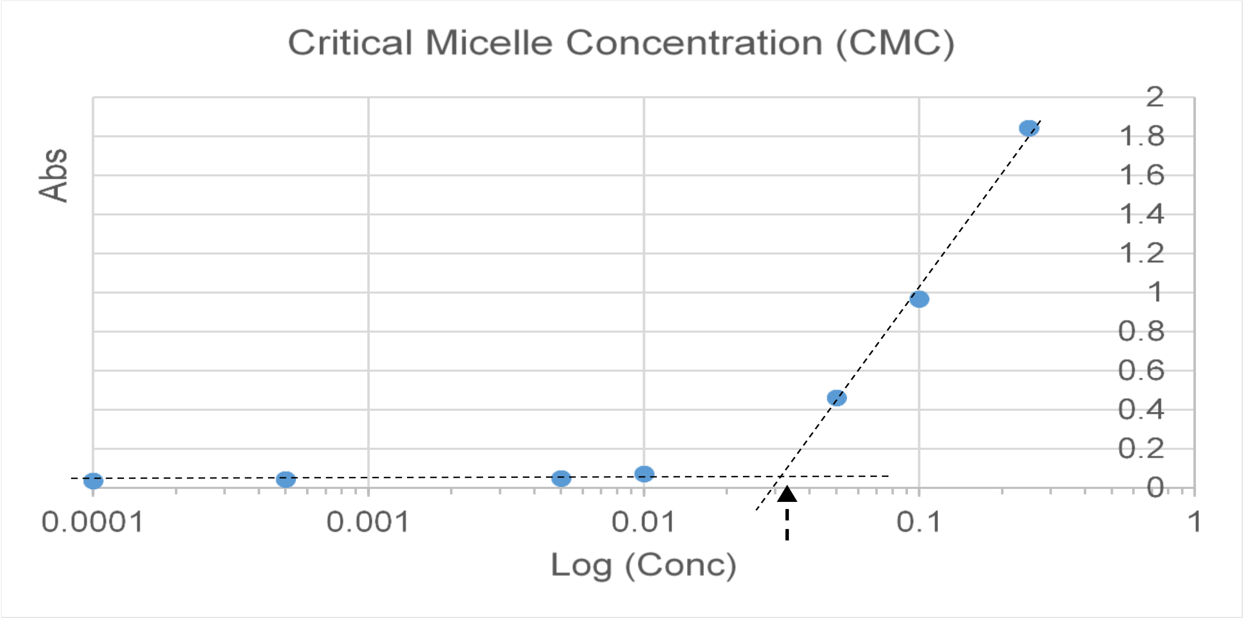

Supplementary Figure S3. Theory mechanism illustrated. a) The mechanism for selective accumulation of saponin molecules in emulsions monolayer. b) Critical micelles concentration (CMC) derived by the plot of absorbance against logarithm of concentration. The CMC value was determined at 0.033 ± 0.001 g/L. A value which further explains the suitability of the proposed mechanism, in terms of competitiveness and comparative advantage.
